# The Role of Information in Managing Interactions from a Multifractal Perspective

**DOI:** 10.3390/e23020148

**Published:** 2021-01-26

**Authors:** Maricel Agop, Stefan Andrei Irimiciuc, Adrian Ghenadi, Luminita Bibire, Stefan Toma, Tudor-Cristian Petrescu, Dorin Vaideanu, Cristina Marcela Rusu, Alina Gavrilut, Decebal Vasincu

**Affiliations:** 1 Department of Physics, Gh. Asachi Technical University of Iași, 700050 Iași, Romania; magop@tuiasi.ro (M.A.); cristina.rusu@tuiasi.ro (C.M.R.); 2 Romanian Scientists Academy, 54 Splaiul Independentei, 050094 Bucharest, Romania; 3 National Institute for Laser, Plasma and Radiation Physics, 409 Atomistilor Street, 077125 Bucharest, Romania; stefan.irimiciuc@inflpr.ro; 4 Department of Industrial Systems Engineering and Management, Faculty of Engineering, University of Bacau, Calea Mărășești 157, 600115 Bacau, Romania; adrian_ghenadi@ub.ro; 5 Department of Environmental Engineering and Mechanical Engineering, Faculty of Engineering, University of Bacau, Calea Mărășești 157, 600115 Bacau, Romania; lbibire@ub.ro; 6 Department of Material Engineering and Industrial Security, Gh. Asachi Technical University of Iași, 700050 Iași, Romania; stefan-lucian.toma@academic.tuiasi.ro; 7 Department of Structural Mechanics, Gh. Asachi Technical University of Iași, 700050 Iași, Romania; tudor.petrescu@tuiasi.ro; 8 Physics Faculty, Alexandru Ioan Cuza University of Iași, 700050 Iași, Romania; dorinvaideanu@student.uaic.ro; 9 Mathematics Faculty, Alexandru Ioan Cuza University of Iași, 700050 Iași, Romania; 10 University of Medicine and Farmacy Grigore T. Popa Iași, 700050 Iași, Romania; decebal.vasincu@umfiasi.ro

**Keywords:** cross-entropy, informational entropy, hydrodynamic model, multifractal attractive and repulsive forces, multifractal Hubble-type law, multifractal Lorentz-type transformation, standard cosmologies

## Abstract

In the framework of the multifractal hydrodynamic model, the correlations informational entropy–cross-entropy manages attractive and repulsive interactions through a multifractal specific potential. The classical dynamics associated with them imply Hubble-type effects, Galilei-type effects, and dependences of interaction constants with multifractal degrees at various scale resolutions, while the insertion of the relativistic amendments in the same dynamics imply multifractal transformations of a generalized Lorentz-type, multifractal metrics invariant to these transformations, and an estimation of the dimension of the multifractal Universe. In such a context, some correspondences with standard cosmologies are analyzed. Since the same types of interactions can also be obtained as harmonics mapping between the usual space and the hyperbolic plane, two measures with uniform and non-uniform temporal flows become functional, temporal measures analogous with Milne’s temporal measures in a more general manner. This work furthers the analysis published recently by our group in “Towards Interactions through Information in a Multifractal Paradigm”.

## 1. Introduction

The evolution of any complex system (e.g., the brain, biological evolution, the Internet, ecosystems, stock markets, economics, and DNA [1,2,3,4]) cannot be predicted only by the behavior of individual entities (structural units) or by the superposition of their behavior. It is determined only by the manner in which the entities relate to the influence of the complex system’s global behavior. Among the significant properties of complex systems are emergence, self-organization, and adaptability [1,2,3,4].

Usually, models used to describe complex system dynamics are based on a combination of basic theories, derived particularly from physics and computer simulations. The description of complex system dynamics implies computational simulations with the use of specific algorithms [5,6,7,8]. The development on the physics standard theory derives from various classes of models:

1. A differentiable class of models based on the usual conservation laws, developed on spaces with integer dimensions [1,2,3];
2. A non-differentiable class of models based on conservation laws, developed on spaces with non-integer dimensions and explicitly written through fractional derivatives [6,7,8,9].

Recently, a new class of models has arisen, based on a multifractal paradigm of motion in the form of the multifractal theory of motion [10,11,12,13,14,15,16,17,18,19]. In these models, supposing that any complex system is assimilated both structurally and functionally to a multifractal object, its dynamics can be described through the motions of the complex system structural units on continuous and non-differentiable curves (multifractal curves). For example, in such a context, for a large temporal scale resolution with respect to the inverse of the highest Lyapunov exponent [20,21,22], the deterministic trajectories of any complex system structural units can be replaced by a collection of potential (virtual) trajectories, while the concept of definite trajectory can be replaced by the one of probability density. Then, the multifractality expressed through stochasticity becomes operational, and correlations of informational entropy—probability density—can be established in the description of the dynamics of any complex system. This means that, instead of working, for example, with a single variable described by a strict non-differentiable function, it is possible to work only with approximations of this mathematical function, obtained by averaging them on different scale resolutions. As a consequence, any variable used to describe complex system dynamics will perform as the limit of a family of mathematical functions, this being non-differentiable for null scale resolutions and differentiable otherwise [10,11,12,13].

In the present paper, using the multifractal hydrodynamic model (Section 2), it is shown that the correlations informational entropy–cross-entropy manages, through a normalized Gaussian, interactions of the attractive and repulsive types (Section 3). Then, classic and relativistic multifractal dynamics become functional in the form of Hubble-type effects, Galilei-type effects, transformation of the generalized Lorentz type, and the invariant metrics of these transformations (Section 4). Correspondences of these models with standard cosmologies are also given in Section 5, while the management of the interactions through harmonic mappings and its correspondences with Milne’s type temporal measures are established (Section 6).

## 2. General Aspects of the Multifractal Hydrodynamic Model and Multifractal Background Matter

Taking into account the diversity of the phenomena that take place in complex systems, it is admitted (as a work hypothesis) that this diversity can be covered by multifractality. Thus, the complex system dynamics will be described through continuous and non-differential curves (multifractal curves). Then, the multifractal theory of motion in its hydrodynamic form becomes functional through the following equations [11,12,13]:

(1) $$\partial_{t}V_{D}^{i}+V^{l}\partial_{l}V_{D}^{i}=-\partial^{i}Q,\;\;\;\;\;\;\;\;\;\;i,l=1,2,3$$

(2) $$\partial_{t}\rho +\partial^{l}\left(\rho V_{D}^{l}\right)=0$$

where *Q* can be expressed as

(3) $$Q=2\lambda^{2}{\left(dt\right)}^{\left[\frac{4}{f\left(\alpha \right)}\right]-2}\frac{\partial_{l}\partial^{l}\sqrt{\rho }}{\sqrt{\rho }}$$

and

(4) $$\partial_{t}=\frac{\partial }{\partial t},\;\;\partial_{l}=\frac{\partial }{\partial X^{l}},\;\;\partial_{l}\partial^{l}=\frac{\partial }{\partial X^{l}}\left(\frac{\partial }{\partial X^{l}}\right),\;\;l=1,2,3$$

In Equations (1–4), t is the non-multifractal time with the role of an affine parameter of the motion curves, $X^{l}$ is the multifractal spatial coordinate, $V_{D}^{i}$ is the multifractal fluid velocity on the differentiable scale resolution, ρ is the states’ density of the multifractal fluid, λ is the structural constant associated with the multifractal–non-multifractal transition, dt is the scale resolution, and f(α) is the singularity spectrum of order α, dependent on the fractal dimension $D_{F}\;$[20,21,22]. Operating with multifractal manifolds instead of monofractal ones, as in Nottale’s scale relativity theory [9], in complex system dynamics, it is possible to identify both the areas of complex system dynamics that are characterized by a certain fractal dimension as well as the number of areas for which the fractal dimensions are situated in an interval of values and classes of universality, even when regular or strange attractors have various aspects.

Equation (1) corresponds to the specific momentum multifractal conservation law, and Equation (2) corresponds to the states’ density multifractal conservation law, while Equation (3) corresponds to the multifractal specific potential as a measure of the multifractalization degree of the motion curves.

Therefore, according to Equations (1)–(3), any complex system structural units are in a permanent interaction with the multifractal medium (multifractal background matter) through the multifractal specific force $-\partial^{i}Q$.

## 3. Informational Entropy–Cross-Entropy Correlations and the Source of Interactions

The informational entropy of a repartition is defined by the relation [23]:

(5) $$H=-\int^{\;}\rho \left(x\right)\ln\rho \left(x\right)dx$$

where ρ(x) is the probability density and x denotes (globally) the random variables of the problem, with dx being the elementary measure of the domain.

This function is a measure of the uncertainty degree in defining the probabilities, in the sense that it is positive, increasing with the increase of uncertainty, in the sense of a broadening of the distribution, which is additive for independent sources of uncertainty. To admit the maximum of the informational entropy in the inference upon the probabilities when only partial information is available is, frankly, the same as admitting the fact that no more knowledge is possible. The obtained distributions must be as such—the ones which derive the least from the real ones—because no restrictive hypothesis is inferred upon the missing information. In other words, such a repartition is made in the largest possible number of measurements.

The partial information, which is available in most cases, is given in the form of the average of a function f(x), or in the form of multiple functions:

(6) $$\bar{f}=\int^{\;}\rho \left(x\right)f\left(x\right)dx$$

Here, it is admitted that $\bar{f}$ is known through a measurement process. Equation (6), together with the relation of the measurement of the distribution of densities, yields

(7) $$\int^{\;}\rho \left(x\right)dx=1$$

These are now constraints which the functional Equation (5) must be subjected to in order to offer the repartition density, corresponding to the maximum of the informational entropy. In this case, Lagrange’s method of undetermined multipliers leads directly to the following exponential repartition:

(8) ρ(x)=exp[−a−bf(x)]

This can be multivariant as well, in cases involving multiple type constraints, as in Equation (6). Let us say that, besides these types of constraints, the following variance is further specified:

(9) $${\left(\Delta f\right)}^{2}=\int^{\;}\rho \left(x\right){\left[f\left(x\right)-\bar{f}\right]}^{2}dx$$

In this case, the nature of the repartition changes to become the Gaussian:

(10) $$\rho \left(x\right)=exp\left[a-bf\left(x\right)-cf^{2}\left(x\right)\right]$$

From the group’s theory point of view [24,25,26], Equation (5) is not invariant, as can be observed. The informational entropy can, however, be rewritten in a manifest invariant form through the introduction of a measure m(x), for which (5) becomes

(11) $$H\left(\rho ,m\right)=\int^{\;}\rho \left(x\right)\ln\left[\frac{\rho \left(x\right)}{m\left(x\right)}\right]dx$$

This function is usually determined through the cross–entropy or variation of the entropy term. Now, the minimization of the entropy variation leads to the same type of repartitions, which differ from one another through the change of the elementary measure of the random variables, as the entropy maximization does:

(12) x→m(x)dx

As such, Equations (8) and (10), become, for example,

(13) ρ(x)=m(x)exp[−a−bf(x)]

and

(14) $$\rho \left(x\right)=m\left(x\right)exp\left[-a-bf\left(x\right)-cf^{2}\left(x\right)\right]$$

From this point of view, the principle of the minimal entropy variation generalizes the principle of maximal informational entropy, with them being identical only in the case in which m(x) is a constant (i.e., operating with uniform repartitions). Because this often is indeed the case, the discussion will revolve around one principle or the other without making any difference between them. Regarding the same aspect, m(x) is presented as a candidate for apriori probabilities, produced with the help of measurable continuous groups; as such, it can be taken as an invariant function on these groups. This is, for example, the case of the SL(2R) group [25,26], which admits unity as an integral invariant function. From a stochastic point of view, it can be said that the variables pertaining to this group are distributed, so the discussion is linked to one of the previously mentioned cases, in which the principle of the minimal variation of entropy is identified with the maximal informational entropy one.

In such a framework, Equation (14) for f(x)≡r and a suitable choice of constants a, b, and c will take the following form:

(15) $$\left(r\right)=\frac{1}{{\left(2\pi \right)}^{\frac{1}{2}}\sigma }exp\left[-\frac{{\left(r-r_{0}\right)}^{2}}{\sigma^{2}}\right]$$

where $r_{0}$ is the average and σ is the variance. From here, using Equation (3), the multifractal specific force results in

(16) $$F\left(r\right)=\frac{-\partial Q}{\partial r}=-\frac{\mu^{2}r_{0}}{\sigma^{2}}\frac{r}{r^{3}}+\frac{\mu^{2}\left(r-r_{0}\right)}{2\sigma^{4}}\frac{r}{r},\;\mu^{2}=2\lambda^{2}{\left(dt\right)}^{\left[\frac{4}{f\left(\alpha \right)}\right]-2}$$

Thus, both the multifractal specific force of an attractive or Newtonian type $F_{N}\left(r\right)=-\frac{\mu^{2}r_{0}}{\sigma^{2}r^{2}}$ and the multifractal force of a repulsive type $F\left(r\right)=\frac{\mu^{2}\left(r-r_{0}\right)}{2\sigma^{4}}$ are natural consequences of the information variation.

## 4. Multifractal Dynamics

In the case where the multifractal inertial effects ∂tV are dominant with respect to the multifractal convective effects (V∇)V, meaning that the condition $\left|\partial_{t}V\right|\gg \left|V\nabla V\right|$ is satisfied, the specific momentum multifractal conservation law from (1) takes the following form:

(17) $$\ddot{r}-\frac{\mu^{2}\left(r-r_{0}\right)}{\sigma^{4}}\frac{r}{r}=-\frac{2\mu^{2}r_{0}}{\sigma^{2}}\frac{r}{r^{3}}$$

where $\ddot{r}=d^{2}r/dt^{2}$ represents the multifractal accelerations.

The above equation can be transformed not only based on the substitution

(18) $$r=R+r_{0}$$

but also based on the constraint:

(19) $$R\gg r_{0}$$

This results in the following equation:

(20) $$\ddot{R}-\frac{\mu^{2}R}{\sigma^{4}}\approx -\frac{2\mu^{2}r_{0}}{\sigma^{2}}\frac{R}{R^{3}}$$

This equation generalizes the results of Newtonian mechanics in the problem of the two bodies and is invariant with respect to the multifractal group of transformations:

(21) $${}^{\prime }=R+\frac{\sigma^{2}}{\mu }V_{0}\sin h\left[\frac{\mu \left(t-t_{0}\right)}{\sigma^{2}}\right]\;t^{\prime }=t,\;\;\;\;\;\;\;\;\;\;\;\;V_{0}=const.$$

The supplementary multifractal accelerations, proportional to the distances from (20), translate the effect of the multifractal ordered component of the motion of the multifractal background matter upon a pair of multifractal objects, with the multifractal constant $\frac{\sigma^{2}}{\mu }$ having time physical dimensions. The multifractal transformation group in (21) is reduced to the multifractal transformation group of the Galilei type for $\frac{\sigma^{2}}{\mu }\to \infty$. Following the lack of local multifractal interaction $\left|\frac{\mu^{2}R}{\sigma^{4}}\right|\gg \left|\frac{2\mu^{2}R}{\sigma^{2}R^{3}}\right|$, the multifractal motion will be described by

(22) $$\frac{d^{2}R}{dt^{2}}-\frac{\mu^{2}R}{\sigma^{4}}\to 0$$

The equation for its solution is given by the expression

(23) $$R=R_{0}\cos h\left[\frac{\mu \left(t-t_{0}\right)}{\sigma^{2}}\right]+\frac{\sigma^{2}}{\mu }V_{0}\sin h\left[\frac{\mu \left(t-t_{0}\right)}{\sigma^{2}}\right]\;R_{0},V_{0}=const$$

Let us say the time of the multifractal motion is far smaller than $\frac{\sigma^{2}}{\mu }$, which is to say

(24) $$\frac{\mu \left(t-t_{0}\right)}{\sigma^{2}}\ll 1$$

In this case, Equation (23) will describe the inertial multifractal motion of the Galilei type:

(25) $$R\approx R_{0}+V_{0}\left(t-t_{0}\right)\;R_{0},V_{0}=const$$

However, if

(26) $$\frac{\mu \left(t-t_{0}\right)}{\sigma^{2}}\gg 1$$

then Equation (23) has a different limit from that in (24), which will be implemented based on the following mathematical procedure. Firstly, Equation (23) will be derived with respect to time; that is,

(27) $$V=\frac{dR}{dt}=\frac{\mu }{\sigma^{2}}R_{0}\sin h\left[\frac{\mu \left(t-t_{0}\right)}{\sigma^{2}}\right]+V_{0}\cos h\left[\frac{\mu \left(t-t_{0}\right)}{\sigma^{2}}\right]$$

Then, $R_{0}$ will be eliminated between Equations (23) and (27), resulting in

(28) $$V=\frac{V_{0}}{\cos h\left[\frac{\mu \left(t-t_{0}\right)}{\sigma^{2}}\right]}+\frac{\mu }{\sigma^{2}}R\tan h\left[\frac{\mu \left(t-t_{0}\right)}{\sigma^{2}}\right]$$

The sought-after limit is of the form

(29) $$V\approx \frac{\mu }{\sigma^{2}}R$$

which corresponds to a multifractal law of the Hubble type.

Now, it is time to make the multifractal transformation of coordinates and time:

(30) $$\tau =\frac{\sigma^{2}}{\mu }\tan h\left[\frac{\mu \left(t-t_{0}\right)}{\sigma^{2}}\right],\;\rho =\frac{R}{\cos h\left[\frac{\mu \left(t-t_{0}\right)}{\sigma^{2}}\right]}$$

upon which the multifractal group in (21) is reduced to the multifractal transformation group of a Galilei type:

(31) $$\rho^{\prime }=\rho +V_{0}\tau \;\tau^{\prime }=\tau ,\;\;V_{0}=const$$

Simultaneously, the following identity is satisfied:

(32) $$\frac{d^{2}\rho }{d\tau^{2}}=\left[\frac{d^{2}R}{dt^{2}}-\frac{\mu^{2}}{\sigma^{4}}R\right]{\cos h}^{3}\left[\frac{\mu \left(t-t_{0}\right)}{\sigma^{2}}\right]$$

From Equations (29) and (32), the result is that in the measure (ρ,τ), a multifractal object, which is acted upon only by the multifractal background (with an almost smooth distribution at the multifractal–non-multifractal transition), has a rectilinear and uniform motion, according to classic prescriptions. To put it differently, the effect of the multifractal background upon the multifractal object, free from any local actions, is entirely compensated; this specifies the fact that the multifractal background dynamic only has a multifractal ergodic component. As a consequence, through the transition from the measure (R,t) to the measure (ρ,τ), the multifractal ordered component (of a hydrodynamic type) is equivalent to the transition from the multifractal class of observational frames to the multifractal class of co-mobile frames. We note that the transitions from the class of observational frames to the class of co-mobile frames is a usual case for the operation of conventional cosmologies.

In what follows from Equation (20), let the multifractal transformation be made:

(33) $$t=t_{0}+\frac{\sigma^{2}}{\mu }ln{\left(\frac{\frac{\sigma^{2}}{\mu }+\tau }{\frac{\sigma^{2}}{\mu }-\tau }\right)}^{\frac{1}{2}},\;R=\frac{\rho }{{\left(1-\frac{\mu^{2}}{\sigma^{4}}\tau^{2}\right)}^{\frac{1}{2}}}$$

which is the inverse of the multifractal transformation in (30). This results in

(34) $$\frac{d^{2}\rho }{d\tau^{2}}=-\chi \frac{1}{{\left(1-\frac{\mu^{2}}{\sigma^{4}}\tau^{2}\right)}^{1/2}}\frac{\rho }{\rho^{3}},\;\;\chi =\frac{2\mu^{2}r_{0}}{\sigma^{2}}$$

Since in the measure (ρ,τ) the multifractal inertial principle of a Galilei type is operational, Equation (34) must be reducible to the multifractal equation of a Newtonian type. As a consequence, the multifractal constant χ must depend on $\frac{\sigma^{2}}{\mu }$ in the followingform:

(35) $$\chi =\chi_{0}{\left(1-\frac{\mu^{2}}{\sigma^{2}}\tau^{2}\right)}^{\frac{1}{2}}=\frac{\chi_{0}}{\cos h\left[\frac{\mu \left(t-t_{0}\right)}{\sigma^{2}}\right]}$$

The multifractal law in (35) corresponds to a decrease of the constant χ with $\frac{\sigma^{2}}{\mu }$.

Until now, a class of multifractal models of a non-relativist type has been developed. Taking into account the relativist amendments, they have to be conceived in a way in which Equation (22), which prescribes the multifractal extended inertial character of the motion of the multifractal object (free of local multifractal actions), must remain unchanged. Then, the mathematical procedure that must be followed becomes obvious: the multifractal transformations in (31) must be substituted with multifractal transformations of a Lorentz type in the measure (ρ,τ). Then, switching from the measure (ρ,τ) to the measure (R,τ), a multifractal transformation group is obtained, which generalizes the Lorentz transformations, which are

(36) $$R^{\prime }={\left(1-\varepsilon^{2}\right)}^{-\frac{1}{2}}\left[\frac{R}{\cos h\;\alpha }+\frac{\gamma \sigma^{2}}{\mu }V_{0}\tan h\;\alpha +\frac{\gamma^{2}}{\gamma +1}\frac{\left(RV_{0}\right)V_{0}}{C^{2}cosh\;\alpha }\right]\;t^{\prime }=t_{0}+\frac{\sigma^{2}}{\mu }ln{\left(\frac{1+\varepsilon }{1-\varepsilon }\right)}^{\frac{1}{2}}\;\gamma ={\left(1-\frac{V_{0}^{2}}{C^{2}}\right)}^{-\frac{1}{2}},\;\;\alpha =\frac{\mu \left(t-t_{0}\right)}{\sigma^{2}},\;\;\varepsilon \equiv \gamma \left[\tan h\;\alpha +\frac{\mu \left(RV_{0}\right)}{{\left(\sigma C\right)}^{2}\cos h\;\alpha }\right]$$

where C corresponds to a limit velocity. These multifractal transformations keep the multifractal metric invariant:

(37) $$ds^{2}=\frac{C^{2}dt^{2}}{{\cos h}^{4}\alpha }\left[1-\frac{1}{C^{2}}{\left(V\cos h\;\alpha -\frac{\mu }{\sigma^{2}}R\;\sin h\;\alpha \right)}^{2}\right]$$

In addition, Equation (22) corresponds to the multifractal geodesic motion in the above-mentioned metric. Finally, the dimensions of the multifractal background can be estimated in this model, admitting that in the measure (ρ,τ), the aggregation of the multifractal background is produced when the multifractal charge of the multifractal Universe becomes null:

(38) $$q={\frac{\chi_{0}}{M_{0}}}^{1/2}\frac{1}{C^{2}}\left[M_{0}C^{2}-\frac{3}{5}\frac{\chi_{0}}{M_{0}}\frac{M_{0}^{3}\mu }{C\sigma^{2}}\right]=0$$

where *M_0_* is the rest mass of the multifractal Universe.

## 5. Correspondences with Standard Cosmologies

Many cosmologic models, although based on the general relativity equations, which obviously include a non-null matter tensor, do arrive at the conclusion that the expansion is of an inertial origin. On the other hand, the empirical law of Hubble did have a provision for an accelerated motion v~r, rather than an inertial one. In a larger sense, an inertial nature can be attributed to an accelerated motion if the equations of motion come from a geodesic motion in a non-Minkowskian metric, for which the curve tensor $R_{\mu \nu }$ is identically null, but the affine connections $\Gamma_{\mu \nu }^{\lambda }$ are non-null. To such a metric, a group of transformations more general than the Lorentz group will correspond, which contains two physical constants: the speed of light and Hubble’s constant. Through considerations linked to the mass and velocity distribution of the Universe’s matter, but also from the fact that the Universe is perceived from a spherical perspective from the origin of the coordinate frame, irrespective of which nucleus of the galaxy the origin lies in, the following equation results:

(39) $$\frac{d^{2}r}{dt^{2}}-\frac{r}{T^{2}}=-G\frac{m_{1}^{0}+m_{2}^{0}}{r^{3}}r$$

where T is the inverse of the Hubble constant *H*, $H=\frac{1}{T}$, G is Newton’s constant, and $m_{1}^{0}$ and $m_{2}^{0}$ are the rest masses of the two bodies (assimilated, for example, with a pair of galaxies).

Now, the correspondence of the multifractal model with the standard cosmological models can be made based on the following considerations:

1. The Universe is assimilated (filled) with a monofractal fluid;
2. The dynamics of the entities of the monofractal fluid are described by continuous and non-differentiable curves (monofractal curves) of a Peano type; the motion trajectories of the entities of the monofractal fluid have the fractal dimension $D_{F}=2$. Thus, the structural constant associated to the monofractal–non-monofractal transition becomes non-dependent of the scale resolution, which is to say
  (40) $$\mu \left(dt,D_{F}\to 2\right)\equiv \lambda$$
3. The correspondence of the monofractal geodesic in Equation (20), submitted to the restriction in Equation (40), with the equation of the motion of two bodies in (39), implies the identities
  (41) $$\frac{\lambda^{2}}{\sigma^{4}}\to \frac{1}{T^{2}},\;\;\frac{2\lambda^{2}r_{0}}{\sigma^{2}}\to G\left(m_{1}^{0}+m_{2}^{0}\right)$$

From here, by choosing

(42) $$2r_{0}\equiv R_{0}\equiv \frac{GM}{c^{2}},\;\;M=m_{1}^{0}+m_{2}^{0}$$

where $R_{0}$ is a gravitational-type specific radius and c is the speed of light in vacuum, this results in

(43) $$\frac{\lambda }{\sigma }=c$$

Moreover, through Equation (41):

(44) σ=cT

In such a context, this results in the following:

1. Hubble’s law, which states
  (45) V≈HR
  This is expressed through Equation (29);
2. The dependence of epoch T of the gravitational constant G, with the form
  (46) $$G=G_{0}{\left(1-\frac{\tau^{2}}{T^{2}}\right)}^{\frac{1}{2}}=\frac{G_{0}}{\cos h\left[\frac{\left(t-t_{0}\right)}{T}\right]}$$
  through Equations (34) and (35) and in the notations
  (47) $$\chi =GM,\;\;\;\chi_{0}=G_{0}M$$

Although the law expressed in (46) corresponds to a decrease of G by T, the difference with respect to the cosmologies of Dirac, Dicke, and others [27] is obvious because $\left(\frac{\dot{G}}{G}\right)=0$ at $t=t_{0}$.

In the same context, the insertion of the relativist amendments has the following implications:

1. The generalization of the Lorentz-type transformations, expressed as
  (48) $$R^{\prime }={\left(1-\varepsilon^{2}\right)}^{-\frac{1}{2}}\left[\frac{R}{\cos h\;\alpha }+\gamma V_{0}T\;\tan h\;\alpha +\frac{\gamma^{2}}{\gamma^{2}+1}\frac{\left(RV_{0}\right)V_{0}}{c^{2}\cos h\;\alpha }\right]\;t^{\prime }=t_{0}+Tln{\left(\frac{1+\varepsilon }{1-\varepsilon }\right)}^{\frac{1}{2}}\;\gamma \equiv {\left(1-\frac{V_{0}^{2}}{c^{2}}\right)}^{-\frac{1}{2}},\;\;\alpha \equiv \frac{\left(t-t_{0}\right)}{T},\;\;\;\;\;\varepsilon \equiv \gamma \left[\tan h\;\alpha +\frac{1}{c^{2}}\frac{\left(RV_{0}\right)}{T\;\cos h\;\alpha }\right]$$
  through Equation (36);
2. The invariant metric, with respect to the transformations in (48), is expressed as
  (49) $$ds^{2}=\frac{c^{2}dt^{2}}{{\cos h}^{4}\alpha }\left[1-\frac{1}{c^{2}}{\left(V\cos h\;\alpha -\frac{R}{T}\sin h\;\alpha \right)}^{2}\right]$$
  through Equation (37);
3. The estimation of the Universe’s dimensions, admitting that in the measure (ρ,τ), the self-structuring of the matter takes place until the gravitational charge of the Universe becomes null; in other words,
  (50) $$q_{G}=\frac{{\left(G_{0}\right)}^{\frac{1}{2}}}{c^{2}}\left[Mc^{2}-\frac{3}{5}G_{0}\frac{M^{2}}{cT}\right]\to 0$$
  through Equation (38).

Thus, a value of the inertial mass M is obtained, which is comparable to the one estimated by Eddington and Milne [27].

## 6. Investigations of Interactions Through Harmonic Mappings and Uniform and Non-Uniform Time Flows

The previous results can also be obtained as harmonic mapping of the usual space and Lobachevsky plane in the Poincaré metric using the Cayley–Klein metrization principle. In such a context, let us consider a circle with the unit radius as an absolute:

(51) $$X^{2}+Y^{2}=1$$

The absolute metric for the interior of Equation (51) is

(52) $$s^{2}=\frac{\left(1-X^{2}\right)dY^{2}+2XYdXdY+\left(1-Y^{2}\right)dX^{2}}{1-X^{2}-Y^{2}}$$

This can be assimilated to the Poincaré metric of the complex plane:

(53) $$ds^{2}=-4\frac{dhd\bar{h}}{\left(h-\bar{h}\right)}=\frac{d{\bar{U}}^{2}+d{\bar{V}}^{2}}{V^{2}}$$

This is done using the following transformations:

(54) $$X=\frac{h\bar{h}-1}{h\bar{h}+1},\;\;Y=\frac{h+\bar{h}}{h\bar{h}+1}\;h=\bar{U}+i\bar{V}=\frac{Y+i{\left(1-X^{2}-Y^{2}\right)}^{\frac{1}{2}}}{1-X}\;\bar{h}=\bar{U}-i\bar{V}$$

From here, through the following substitutions are made:

(55) $$X=e\cos\;\bar{\Omega },\;\;Y=e\sin\bar{\Omega },\;\;e=\tanh\psi$$

Thus, Equation (53) takes the form

(56) $$s^{2}={\left(\frac{de}{1-e^{2}}\right)}^{2}+\frac{e^{2}}{1-e^{2}}{\left(d\bar{\Omega }\right)}^{2}={\left(d\psi \right)}^{2}+\sin h^{2}\psi {\left(d\bar{\Omega }\right)}^{2}$$

The complex parameters h and $\bar{h}$ from Equation (54) now have a direct connection with the classic theory of Newtonian-type potentials (for details, see [12,13,28,29,30]) based on harmonic mappings [31]. In order to prove this, we need to rewrite h and $\bar{h}$ in the terms (χ,Ω). This results in

(57) $$h=i\frac{\cosh\chi -\sinh\chi e^{-i\bar{\Omega }}}{\cosh\chi +\sinh\chi e^{-i\bar{\Omega }}},\;\;\chi =\frac{\psi }{2}$$

Equation (57) represents the harmonic mappings from the usual space to the Lobacevsky plane—having the metric expressed in (53)—as long as χ (and thus ψ) are solutions of a Laplace-type equation for the free space. Indeed, the issue of harmonic mappings from the usual space to the hyperbolic plane is described through the stationary values of the Lagrangean:

(58) $$L=-4\frac{\nabla h\nabla \bar{h}}{{\left(h-\bar{h}\right)}^{2}}$$

This is specific to the variational problems

(59) $$\delta \int^{\;}Ld\tau =0$$

where ∇ corresponds to the gradient and dτ corresponds to the infinitesimal volume. From here, the field equations result in

(60) $$\left(h-\bar{h}\right)\nabla \left(\nabla h\right)=2{\left(\nabla h\right)}^{2}$$

which admits Equation (57) as a solution. Of course, along with Equation (60), the field equations for the complex conjugate $\bar{h}$ are also satisfied.

On the other hand, the Minkowski-type metric is written in the form

(61) $$Z^{2}=c^{2}T^{2}+W^{2},\;W=\frac{c^{2}}{\alpha }$$

where α is the magnitude of acceleration. This offers the possibility for explaining the Z=const. constraint for any entity of the complex system engaged in a hyperbolic motion. In this case too, Equation (61) can be interpreted as absolute in the coordinates

(62) $$x=\frac{W}{Z},\;\;y=\frac{cT}{Z}$$

Indeed, for

(63) $$h=\frac{y+i{\left(1-x^{2}-y^{2}\right)}^{\frac{1}{2}}}{1-x}$$

or explicitly

(64) $$h=\frac{cT\pm i{\left(Z^{2}-W^{2}-c^{2}T^{2}\right)}^{\frac{1}{2}}}{Z-W}$$

the metric in (53) is obtained.

The field equation in (60), along with

(65) h=u+iv

is equivalent with the pair of real equations

(66) $$v\nabla^{2}u-2\nabla u\nabla v=0\;v\nabla^{2}v-{\left(\nabla v\right)}^{2}+{\left(\nabla u\right)}^{2}=0$$

where the differential operators are considered in a three-dimensional arbitrary metric.

As can be observed, the constraint T=0 implies the following situation:

(67) $$h=iv\Leftrightarrow v=\frac{{\left(Z^{2}-W^{2}\right)}^{\frac{1}{2}}}{Z-W}$$

Here, the fields are characterized by the second equation from (66). Thus, through $v=e^{\phi }$, the equations in (66) are reduced to the Laplace equation

(68) △ϕ=0

Equation (68) admits, for a spherical symmetry, the solution

(69) $$\phi \left(R\right)=C_{1}-\frac{C_{2}}{R},\;\;\;\;C_{1},\;C_{2},\;=const.$$

This is a solution which, through Equation (67) and an adequate choice of the integration constants, becomes

(70) $$\frac{Z+W}{Z-W}=exp\left(-\frac{K}{R}\right),\;\;K=const.$$

Equation (70) can be used to extract the coordinate W in the form

(71) $$W=-Z^{2}\tanh\left(\frac{K}{R}\right)$$

From here, at the limit $\frac{K}{R}\to 0$, we obtain

(72) $$W=-\frac{ZK}{R}$$

Alternatively, by means of the second relation in (61), we get

(73) $$\alpha =-k^{2}R,\;k^{2}=\frac{ZK}{c^{2}}$$

As such, at T=0, α can be assimilated to an acceleration in a radial oscillatory motion or, taking into account Equation (73), it can be assimilated to a centripetal acceleration in a uniform circular motion.

The utilized variational principle which gives the above-mentioned result (i.e., Equation (59)) is obtained from the metric in (53), which is invariant when related to a certain transformation group: the group SL(2R), to be precise (for details, see [11,12,13,28,29,30,31]). Now, if the functionality of the equivalence principle is admitted for an arbitrary manifold point, the coordinate W represents the intensity of the fields, while the previously mentioned group represents the transition between the various fields which act in that point. The appearance of the simultaneous action of these fields is a rotational motion characterized by the centripetal force in Equation (73) to the initial moment, far from the common center of the field sources.

Thus, by substituting the principle of simultaneous independent actions with the a priori invariance of the field action, with respect to a certain group, it is possible to develop complex system dynamics theories free of any inherent contradictions found in standard theories [27].

Such a unitary operational procedure (built on harmonic mappings) allows the definition of two temporal measures (which shall not be postulated): one characterized by a uniform time flow and specific descriptive dynamics in the global frame (on multifractal background matter and imposed by a uniform circular type motion) and the other one characterized by a non-uniform time flow and specific to the descriptive dynamics in the local frame (on the entities of the multifractal background matter and imposed by Newtonian-type motion). From such a perspective, the functionality of the multifractal group of the *SL(2R)* type (as an invariant group of the hyperbolic metric) allows correlations of local–global dynamics through the synchronization of the previously mentioned temporal measures. These can be analogues with Milne’s temporal measures, the temporal measure of the intergalactic scale (specific to the descriptive in the interior of the galaxy and the temporal measure of the extragalactic scale, which is specific to the descriptive in the observable universe, i.e., cosmological time).

**Figure 1:**
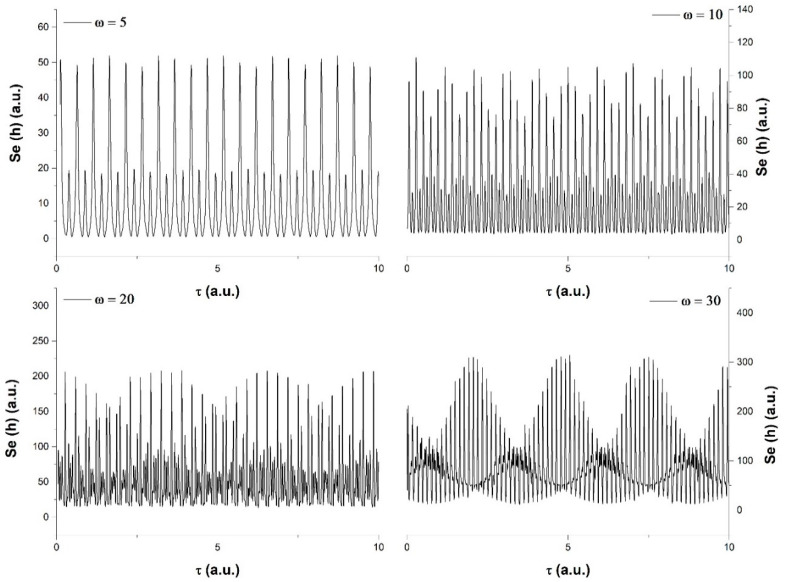
Scale resolution effect on the *h* function time series for a linear internal time flow using ωτ

In Figure 1, we have represented the dynamics of the system described through Equations (57) or (64), considering a usual temporal expansion of the type $\bar{\Omega }=\omega \tau$ for various harmonic-type resolution scales (ω = 5, 10, 20, 30). The evolution depicted here is similar to the other iterations of our model, which can be found in [11,32] where, for a linear flow of time, the natural background state of our system is a period double state. The increase in the harmonic resolution scale leads to an increase in the oscillation frequency, with clear modulation overlapping the basic state. The modulation appears as a result of the interaction between the complex systems and the fractal background medium. The fractal background infringes on the natural expansion of the system. The imposed dynamic and the natural expansion will converge on the surface area which separates the systems, thus showing signatures from both sources. A further increase leads into a more dominant modulation state, which reflects the change in balance between the main driving mechanism that dictates the evolution of the system. In these harmonic scale resolutions (ω = 20, 30), the system is clearly more influenced by the fractal background matter which, according to our reports from [32,33,34], usually leads to a quasi-chaotic state. The role of information at this scale was investigated in detail and reported by our group in [35,36,37,38].

**Figure 2:**
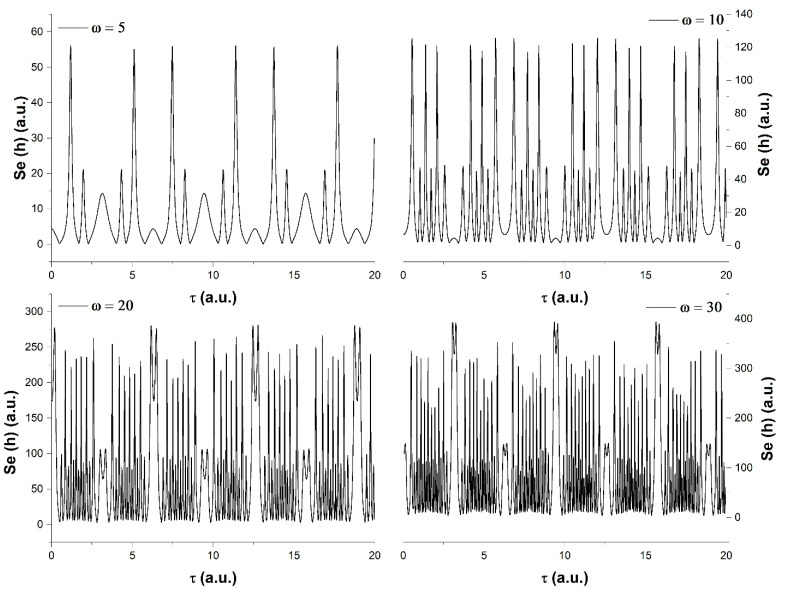
Scale resolution effect on the *h* function time series for a nonlinear internal time flow using *cn (τ; 0.1)*.

By changing the nature of the time flow and choosing a *cn (τ*; 0.1)-type evolution for the internal local time, we observed that there were some aspects that tranced the choice of time. The basic oscillatory signature is seen here again as period doubling (Figure 2). The transition here as the harmonic scale resolution is augmented through intermittences, coupled with an increase of the oscillation frequency. It can also be seen that between the oscillation packs, there is a single peak transition which increases its amplitude, and it becomes visibly more complex at ω = 20, 30, where secondary overlapping oscillation can be seen for that region.

**Figure 3:**
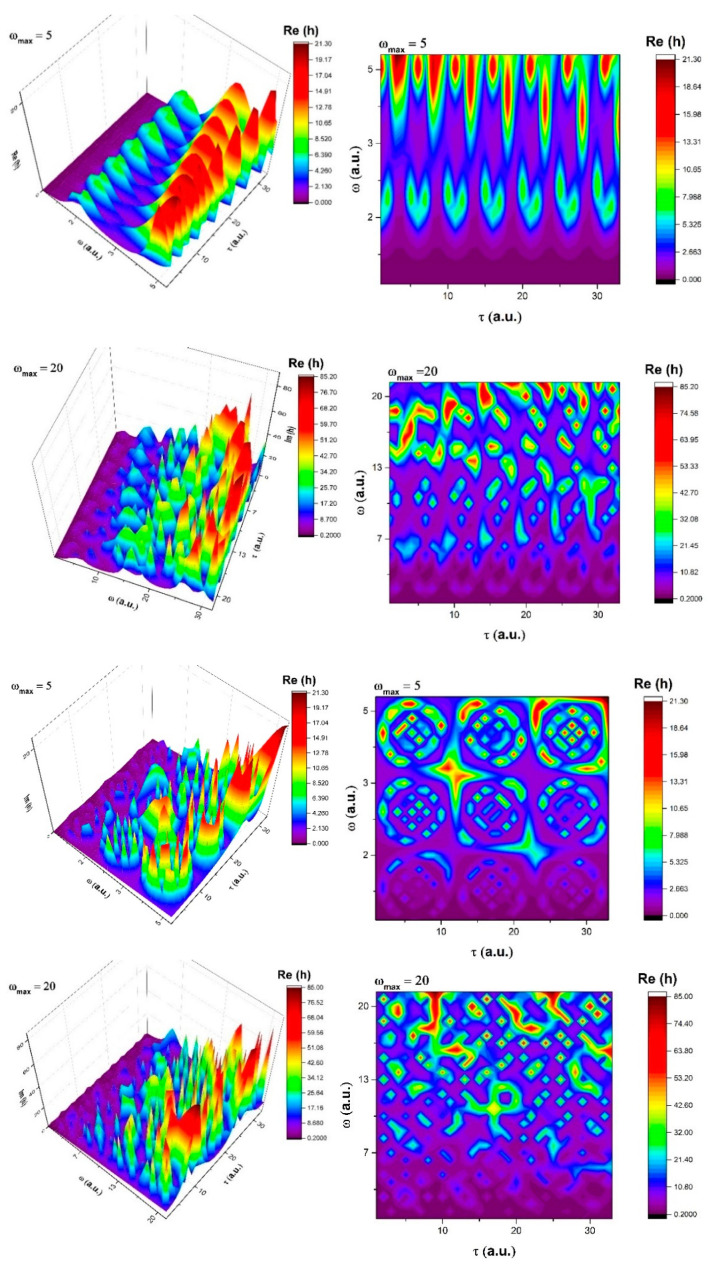
3D representation and contour plot for Re(h) at ω_max_ = 5 and 20 for a linear internal time flow using *cn* (*τ*; 0.1) and ωτ

Let us now compare the two types of dynamics at a global scale by representing the Re(h) for ω = 5 in the two selected cases for the time flow. The results can be seen in Figure 3. We can see considerably different behaviors with the linear flow, representing the structuring and an increase in oscillation amplitudes with the flow of time. The non-linear flow of time presents an interesting structure where we see multiple maxima: two fixed (one at τ = 2–2.5 and one at τ = 4–5) and a shifting structure that evolves from τ = 5 to τ = 3. This shift corresponds to the intermittent changing structure seen in Figure 3, which represents the transition between the packets of oscillations. The change of the maximum harmonic scale resolution from 5 to 20 reveals a transition toward a quasi-chaotic movement for the linear time flow, with strong overlapping of the structuring seen at lower values and multiple structuring, but with a clear lower chaoticity of the harmonic maps. These results are essential in understanding different time flows for inner (local) and exterior (global) time, as it is clearly seen here that the predilection of chaos exists in both cases, but it is explicitly shown only for external linear time while, for inner nonlinear time, there is bigger inertia against the transition toward chaos.

## 7. Conclusions

A review of the multifractal theory of motion in its hydrodynamic form is presented, with the focus on presenting the conservation laws and the explicit form of the multifractal specific potential. The correlation of informational entropy–cross-entropy manages—through a multifractal specific potential—the normalized Gaussian with a spherical symmetry for both attraction and repulsion. Multifractal classical dynamics associated to attractive and repulsive forces imply multifractal Hubble-type effects, multifractal Galilei-type effects, and dependencies of some interaction’s constants with multifractality degrees. The insertion of the relativistic amendments in the attractive and repulsive dynamics implies not only multifractal transformations of generalized Lorentz-type, multifractal metrics invariant to these transformations, but also an estimation of the dimension the multifractal Universe, admitting that, in a particular space–time measure, the aggregation of the multifractal background matter is produced when the multifractal charge of this Universe becomes null. By assimilating the universe with a monofractal fluid, whose entities are moving on Peano-type curves, some correspondences of our model with standard cosmologies were analyzed as well. Attractive and repulsive interactions were also obtained as harmonic mapping between the usual space and the hyperbolic space with the Cayley–Klein metrization principle. Two mechanisms with uniform and non-uniform temporal frames become functional. Finally, for the dynamics of the entities of multifractal background matter on Peano-type curves, it is shown that these temporal measures become analogous with Milne’s temporal measures.
